# Inhibition of cellular NEDDylation partially alters the MHC class I immunopeptidome

**DOI:** 10.1093/jimmun/vkag258

**Published:** 2026-10-10

**Authors:** Hooman Yari, Konstantin Divilov, Dylan T Fahey, Sofia A Arola, William H Hildebrand, Brian P Dolan

**Affiliations:** Microbiology & Immunology, The University of Oklahoma Health Sciences Center, Oklahoma City, OK, United States; Center for Quantitative Life Sciences, Oregon State University, Corvallis, OR, United States; Carlson College of Veterinary Medicine, Biomedical Sciences, Oregon State University, Corvallis, OR, United States; Carlson College of Veterinary Medicine, Biomedical Sciences, Oregon State University, Corvallis, OR, United States; Microbiology & Immunology, The University of Oklahoma Health Sciences Center, Oklahoma City, OK, United States; Carlson College of Veterinary Medicine, Biomedical Sciences, Oregon State University, Corvallis, OR, United States

**Keywords:** MHC class I immunopeptidome, tumor antigen presentation, ubiquitin ligase

## Abstract

Determining how chemotherapeutic drugs alter the MHC class I–associated immunopeptidome is essential for designing rational combinatorial therapies to enhance antitumor CD8^+^ T-cell activity, such as immune checkpoint inhibitor Abs. We investigated the impact of a first-in-class inhibitor of cellular NEDDylation, MLN4924, on self-peptide presentation using 2 human tumor cell lines: HeLa cells secreting the human MHC class I protein HLA-A*11:01 and the glioblastoma cell line U87-MG. MLN4924 indirectly inactivates the cullin-RING ligases (CRLs), a large family of E3 ubiquitin ligases responsible for targeting numerous substrates for degradation by the ubiquitin-proteasome system. MLN4924 treatment prevented cell division and altered the transcriptome of HeLa cells in a predictable manner. However, MLN4924 treatment did not alter the cell-surface levels of MHC class I proteins. An analysis of the immunopeptidome and the source proteins, which yielded peptides for presentation, revealed some unexpected findings. Greater than 50% of the peptides presented, and more than two-thirds of the source proteins from which peptides were derived, remained unaltered after MLN4924 treatment. Furthermore, alterations to the transcriptome could not predict the observed changes to the immunopeptidome. Of the approximately 100 CRL substrates that were presented by HLA-A*11:01, most were presented independent of MLN4924 treatment. Alternative degradation pathways and the presence of intron sequences in the specific mRNA transcripts may help explain how these proteins yield peptides for Ag presentation. In summary, MLN4924 treatment only partially affects the immunopeptidomic profile, suggesting it could be used in combinatorial cancer immunotherapies.

## Introduction

CD8^+^ T cells survey the cells of the body to determine if they are infected or transformed. The disease state of cells is communicated to cytotoxic T cells using the MHC class I Ag processing and presentation pathway. In brief, proteins synthesized and subsequently degraded into short peptide sequences within the surveyed cells are transported into the endoplasmic reticulum (ER) and bind to MHC class I proteins. The peptide–MHC class I complex (pMHC) is then transported to the cell surface where the T-cell receptor (TCR), which is unique to each CD8^+^ T cell, can attempt to bind to the pMHC. Strong binding of the TCR to the pMHC indicates to the T-cell that its specific Ag is present in the target cell and will signal to the T cell to initiate its cytotoxic action to eliminate the cell. If the TCR fails to bind to the pMHC complex, the cell is spared. The antigenic peptide, therefore, is the lynchpin in cytotoxic T-cell reactions, and understanding how Ag peptides are generated is essential to alter immune outcomes in diseases involving CD8^+^ T cells.

Cellular proteins are often degraded by the ubiquitin-proteasome system. In a stepwise process, the protein destined for proteasomal degradation is covalently modified by the addition of ubiquitin, a small, 8-kDa protein that can form chains when attached to the protein substrate. Although the outcomes of protein ubiquitylation can be varied, often the covalent modification by ubiquitin is a signal for proteasomal degradation. Many reports over the years have indicated that both a functional ubiquitin-conjugation system and proteasome are necessary for Ag presentation.^1–11^ Loss of either system can result in loss of total MHC class I at the cell surface and specific pMHC complexes.

The covalent modification of a protein with ubiquitin is carried out by a group of enzymes collectively known as E3 ubiquitin ligases. There are more than 600 genes encoding different E3 ubiquitin ligases in the human genome. One important family of E3 ubiquitin ligases, the cullin-RING ligases (CRLs), are multiprotein enzymes that are controlled by another post-translational modification (PTM) similar to ubiquitination. The cullin protein component of the CRL must be modified by the addition of neural precursor cell–expressed developmentally downregulated protein 8 (NEDD8), a ubiquitin-like protein.^12^ NEDDylation of the cullin protein leads to a conformational change of the CRL and brings the ubiquitin-containing E2 component of the CRL near the enzyme’s substrate, facilitating transfer of ubiquitin to the substrate.^13^^,^^14^ The different combinations of cullin proteins, RING-finger ligases, and E2 conjugating enzymes can generate more than 100 different CRL ligases; however, all can be inactivated by preventing cullin NEDDylation.

The development of chemical inhibitors of cellular NEDDylation has greatly expanded not only our knowledge of the functions of NEDD8 but also has provided a novel chemotherapeutic agent for the treatment of cancers.^15–17^ MLN4924/TAK-924 (pevonedistat) was the first described inhibitor of the NEDD8-activating enzyme NAE1,^18^ and treatment of cells with MLN4924 revealed a disruption of the cell cycle and accumulation of CRL substrates within the cell. The drug is in multiple clinical trials for a variety of cancers.^19^^,^^20^ A second-generation NAE1 inhibitor, TAS4464, which is more potent than MLN4924, has recently been described.^21^^,^^22^

Immunotherapies for the treatment of cancer, such as immune checkpoint inhibitors (ICIs)^23^ and adoptive transfer of tumor-infiltrating lymphocytes,^24^ rely on the ability of T cells to recognize tumor cells via MHC class I Ag presentation, and future oncology treatments will almost certainly combine immunotherapies with existing and novel chemotherapies. Loss of MHC class I expression or tumor Ag presentation can alter immunotherapy outcomes^25–27^; therefore, careful attention must be paid to how the chemotherapeutic agents affect the MHC class I Ag presentation pathways. Agents that inhibit MHC class I Ag presentation or greatly alter the immunopeptidome of cancer cells would make poor partners for therapies designed to enhance antitumor T-cell function.

Given both the interest in developing inhibitors of cellular NEDDylation and the lack of knowledge on how different E3 ligases may affect MHC class I Ag presentation, we sought to characterize how MLN4924 treatment of tumor cells would affect both overall MHC class I Ag presentation and the composition of the immunopeptidome. Here we report that MLN4924 treatment of HeLa cells did not affect overall MHC class I levels. Mass spectrometry analysis of the immunopeptidome of both HLA-A*11:01 proteins secreted from HeLa cells and total HLA class I molecules on U87-MG glioblastoma cells demonstrated that most of the presented peptide identities and corresponding source proteins remained unchanged after MLN4924 treatment.

## Materials and methods

### Cell lines and Abs

HeLa cells and U87-MG cells were grown in DMEM (Gibco) supplemented with 7.5% FBS (Atlanta Biologicals), and Glutamax (Gibco) and were cultured at 37 °C in 5% CO_2_. HeLa cells expressing sHLA-A*11:01 tagged with the very-low-density lipoprotein receptor (VLDLr) purification epitope tag SVVSTDDDLA, described elsewhere,^28^ were grown in RPMI 1640 medium supplemented with 10% FBS. The following Abs were used in this study: pan HLA class I mAb W6/32 from HB-95 (ATCC) and purchased from Leinco; mouse mAb clone AC-15 anti–β actin (Sigma-Aldrich); mouse mAb clone FK2 anti-ubiquitin (Enzo Life Sciences); mouse mAb clone 195720 anti-p21 (R&D Systems); mouse mAb anti–HIF-1α (BD Transduction Laboratories); rabbit mAb clone D6L4J anti–ETS-2 (Cell Signaling Technology); rabbit mAb clone D10F11 anti-CDT1 (Cell Signaling Technology); rabbit mAb clone D1Z9C anti-NRF2 (Cell Signaling Technology); and mouse IgG-6A6 Ab against rat VLDLr (ATCC CRL-2197). The following infrared secondary Abs were from LI-COR and used for Western blot detection: goat anti-mouse IR Dye 800 and goat anti-rabbit IR Dye 680. MLN4924 and MLN7243 were from MedChemExpress and dissolved in DMSO at a concentration of 10 mM.

### Drug treatments

For analysis of total cell-surface MHC class I, cell division, and cell size, cells were treated with indicated concentrations of MLN4924 for 48 h prior to analysis. For transcriptomic analysis, HeLa cells were treated for approximately 2 d with 1 µM MLN4924 or DMSO before cell collection. For Western blot analysis, HeLa cells were treated with 5 µM MLN4924 or 10 µM MLN7243 for 5 h before cell harvesting.

### Determination of the concentration of soluble HLA in supernatants

Concentration of soluble HLA-A*11:01 in the cell culture supernatants was measured by sandwich ELISA using the anti-VLDLr mAb as the capture Ab and W6/32 Ab for detection of soluble HLA.

### Transcriptome analysis

Total RNA was isolated from HeLa cells using Nucleospin RNA Plus kits from Macherey-Nagel, following the manufacturer’s recommended procedure. RNA sequencing (RNA-Seq) libraries were prepared using the NEBNext Poly(A) mRNA Magnetic Isolation Module (New England Biolabs) followed by the NEBNext Ultra II Directional RNA Library Prep Kit for Illumina (New England Biolabs) and sequenced using a P1 flow cell (1 × 100 bp) on a NextSeq 2000 system (Illumina). The nf-core/rnaseq, v3.14.0, pipeline^29^ was used to perform quality control, trim reads (fastp), align reads (STAR) to the GENCODE release 45 human genome annotation,^30^ and quantify gene expression (Salmon). The nf-core/differential abundance, v1.4.0, pipeline was used to test genes with at least 10 counts in at least 2 samples for differential gene expression between treatments (DESeq2). Significantly differentially expressed genes were processed through the g: Profiler web server^31^ to test for Kyoto Encyclopedia of Genes and Genomes (KEGG) pathway enrichment. To quantify intron abundance for genes, AGAT, v1.4.1,^32^ was used to annotate introns in the GENCODE genome annotation, and the nf-core/rnaseq pipeline was run as explained except counts were based on read alignment to introns rather than exons.

### Soluble HLA collection and peptide isolation

To collect soluble HLA (sHLA) from control or MLN4924-treated cells, cells were cultured in complete medium supplemented with MLN4924 at a concentration of 1 µM in T-150 cell culture flasks. The medium was harvested and discarded after 24 h. Fresh complete medium containing MLN4924 was then added to the flasks, and the supernatant was collected to isolate sHLA. This process was repeated every 3 d for 2 wk to collect the supernatant containing sHLA produced by MLN4924-treated transfected cells. The sHLA-peptide complexes were purified from cell supernatants by affinity chromatography using the anti-VLDLr Ab coupled to CNBr-activated Sepharose 4 Fast Flow (Amersham Pharmacia Biotech). Peptide was released from class I heavy and light chains by denaturing of 100 µg of the purified sHLA with 10% acetic acid followed by heating at 76 °C for 10 min.

### Generation of W6/32 immunoaffinity column and total HLA purification

Purification of total HLA followed the general protocol described by Purcell et al.^33^ An immunoaffinity column was generated by coupling 5 mg of W6/32 Ab (Leinco) to 2 mL of bed volume Protein A Sepharose beads (Repligen), which first had been washed with PBS, and by gentle mixing for 1 h at room temperature. Coupled Abs and beads were loaded into a 2-mL Econo-Column (Bio-Rad) and washed with 10 column volumes (c.v.) of borate wash buffer (0.05 M boric acid, 0.5 M KCl, and 4 mM NaOH), followed by 10 c.v. of 0.2 M triethanolamine (TEA), pH 8.2. To crosslink the Abs to the Protein A Sepharose beads, 5 c.v. of 40 mM dimethyl pimelimidate (DMP) (Sigma-Aldrich), freshly prepared by dissolving 250 mg of DMP-2HCl in 22 mL of 0.2 M TEA and adjusting the final pH to 8.3, was added to the column. The column was capped, and cross-linking was allowed to proceed for 1 h at room temperature. Cross-linking was terminated by washing with 10 c.v. of cold 0.2 M Tris at pH 8.0, and unbound Ab was stripped away with 10 c.v. of 0.1 M citric acid at pH 3.0. Finally, PBS, 7.4 pH, was applied to the column until the pH of the flow through was 7.4. Approximately 1 × 10^8^ U87-MG cells were cultured in the presence or absence of 1 µM MLN4924 for 48 h. Cells were harvested and lysed in lysis buffer (50 mM Tris, pH 8.0, 150 mM NaCl, 0.5% IGEPAL CA-630 [Sigma-Aldrich], and Roche cOmplete EDTA-free Protease Inhibitor tablet) at a concentration of 2 × 10^7^ cells/mL for 15 min on ice. Lysates were then centrifuged at 2,000*g* at 4 °C for 5 min, and the supernatant was applied to a Nucleospin gDNA removal column (Macherey-Nagel), centrifuged at 11,000*g* for 1 min, and finally filtered through a 0.2 μm HT Tuffryn membrane. The clarified lysates were precleared by addition to an Econo-Column packed with 1 mL of PBS-washed Protein A Sepharose beads and washed with 3 c.v. lysis buffer. The precleared lysate and wash were applied to the W6/32 immunoaffinity column, which had been prewashed with 10 c.v. lysis buffer. The flow through was collected and reapplied to the column. The column was then washed with 10 c.v. of wash buffer 1 (0.005% IGEPAL CA-630, 50 mM Tris at pH 8, 150 mM NaCl, 5 mM EDTA, 100 μM PMSF, and 1 μg/mL pepstatin A), followed by 10 c.v. wash buffer 2 (50 mM Tris at pH 8, and 150 mM NaCl), 10 c.v. of wash buffer 3 (50 mM Tris at pH 8, 450 mM NaCl), and 10 c.v. of wash buffer 4 (50 mM Tris, pH 8). Finally, the MHC-peptide complexes were eluted with 5 c.v. of 10% acetic acid and stored at −80°C.

### Fractionation of the HLA/peptide mixture by reverse-phase HPLC

The mixture of the peptides and sHLA isolated from HeLa cells or peptides and cell-retained HLA isolated from U87-MG cells eluted from the affinity column was further purified and fractionated by reverse-phase HPLC. First, the eluates were dried under vacuum using a CentriVap concentrator (Labconco). The solid residues were dissolved in 10% acetic acid in water solution and fractionated over a 150-mm-long Gemini C18 column, with pore size of 110 Å and particle size of 5 μm (Phenomenex), using a Shimadzu Nexera instrument (Shimadzu Scientific Instruments). An acetonitrile (ACN) gradient was run at pH 2 using a 2-solvent system. Solvent A contained 2% ACN in water, and solvent B contained 5% water in ACN. Both solvent A and solvent B contained 0.1% trifluoroacetic acid. The column was pre-equilibrated at 2% solvent B. The sample was loaded on the column over 18 min using a solvent system composed of 2% solvent. Then a 2-segment gradient was run at a flow rate of 160 µL/min: 4% to 40% solvent B for 40 min, followed by 40% to 80% solvent B for 8 min. Fractions were collected in 2-min intervals using a Gilson FC 203B fraction collector, and the UV absorption profile of the eluate was recorded at 215-nm wavelength.

### Nano–liquid chromatography–tandem mass spectrometry analysis

Nano–liquid chromatography–tandem mass spectrometry (LC–MS/MS) analysis was performed on isolated peptides using a previously described method.^34^ Briefly, peptide-containing HPLC fractions were dried and resuspended in 10% acetic acid, 2% ACN, and iRT peptides (Biognosys) as internal standards, and then analyzed individually by LC–MS/MS. Samples were injected onto either a Sciex TripleTOF 5600 mass spectrometer coupled to an Eksigent nanoLC 415 system (AB Sciex), or a Sciex ZenoTOF 7600 (AB Sciex) coupled to a Waters Acquity M-Class UPLC Instrument (Waters). Instrument-specific LC gradients and information-dependent acquisition methods were used, and precursor ions were fragmented by collision-induced dissociation with dynamic collision energy.

For the TripleTOF 5600 platform, peptides were separated on ChromXP C18 trap and analytical columns using a 2-solvent system (0.1% formic acid in water and 0.1% formic acid in 95% ACN). After loading at 5 μL/min, separation was performed at 300 nL/min with linear gradients of 10% to 40% ACN over 70 min followed by 40% to 80% ACN over 7 min. Survey scans (300 to 1,250  mass-to-charge ratio [*m*/*z*]) were acquired for 0.25 s, and up to 22 precursor ions (charge state 2–5, ≥200 cps) were selected for MS/MS, with dynamic exclusion of 30 s after 3 occurrences.

For the ZenoTOF 7600 system, peptides were separated on NanoEase C18 trap and analytical columns, using a similar solvent system with a shorter, multistep gradient (2% to 40% ACN over 40 min, followed by 40% to 80% over 7 min) at 500 nL/min. Survey scans (350 to 1,250 *m*/*z*) were acquired for 0.25 s, and up to 50 precursor ions (charge state 2–4, ≥100 cps) were selected for fragmentation with a 0.05 s accumulation time and 12 s dynamic exclusion. Data were visualized and processed using PeakView software, version 1.2.0.3, or Sciex OS software, version 4.0.0.8559 (AB Sciex).

### Peptide data analysis

Peptide sequences were identified using PEAKS Studio 11.5 software (Bioinformatics Solutions) at a precursor mass error tolerance of 30 ppm and a fragment mass error tolerance of 0.02 Da. A database comprising Swiss-Prot *Homo sapiens* (taxon identifier 9606) (https://www.uniprot.org) and iRT peptide sequences was used as the reference for the database search. Variable PTMs including acetylation, deamidation, pyroglutamate formation, oxidation, sodium adducts, phosphorylation, and cysteinylation were included in database search. Identified peptides were further filtered at a peptide false discovery rate of 1% using PEAKS decoy-fusion algorithm and, after removing PTMs from peptide sequences, they were assigned to HLA alleles by submitting to MHCMotifDecon 1.2 available via https://services.healthtech.dtu.dk/services/MHCMotifDecon-1.2 (Denmark Technical University).^35^ For unsupervised clustering of peptides, the sequences with 8 to 13 amino acid length were submitted to GibbsCluster 2.0 via https://services.healthtech.dtu.dk/services/GibbsCluster-2.0/ (Denmark Technical University).^36^ The potential mutated peptides were found by homology search of de novo sequences against the aforementioned database.

### Western blot

Cell lysates were prepared by lysing HeLa cells at a concentration of 2 × 10^7^ cells/mL in RIPA buffer containing cOmplete Protease Inhibitor (Roche) for 15 min on ice. Lysates were centrifuged at high speed for 5 min at 4 °C to pellet insoluble material. Lysates were then mixed with Bolt Sample Buffer (Invitrogen) and DTT (1 mM final) and boiled for 10 min. Proteins were separated by SDS-PAGE using 4% to 12% Bolt Bis-Tris gels (Invitrogen) and blotted onto nitrocellulose membranes (Invitrogen). Membranes were blocked in 5% nonfat milk in TBS-T buffer for 1 h and stained with primary Abs overnight. Membranes were washed and appropriate secondary Abs were added for 1 h before analysis on an Azure c600 imager equipped with infrared detection systems.

### Flow cytometry

Approximately 10^5^ cells were stained with APC-coupled W6/32 mAb for 30 min at 4 °C diluted in HBSS supplemented with 0.5% BSA. Cells were washed twice and resuspended in buffer for analysis on an Accuri C6 flow cytometer. The mean fluorescent intensity of 10,000 live cells (determined by forward scatter/side scatter analysis) was recorded in triplicate.

### Statistical analysis

GraphPad Prism software, version 10.5 (www.graphpad.com), was used to fit size-distribution data in the log normal distribution model to calculate peptide length geometric means and other statistical analyses.

## Results

### MLN4924 inhibits cell division and degradation of CRL substrates

To confirm that MLN4924 inhibited CRL function in our system, we treated ∼250,000 HeLa cells with 0.5, 1.0, or 5.0 µM MLN4924 for 48 h. As shown in Fig. 1A, the total cell number remained relatively unchanged over the 2-d drug treatment, whereas untreated cells underwent ∼1.5 rounds of cell growth, consistent with a doubling period of ∼24 h for HeLa cells. There was no evidence of cell death during drug treatment, indicating that MLN4924 arrested growth. We also noted that cells increased in size, as determined by forward light scatter analysis (Fig. 1B).

**Figure 1:**
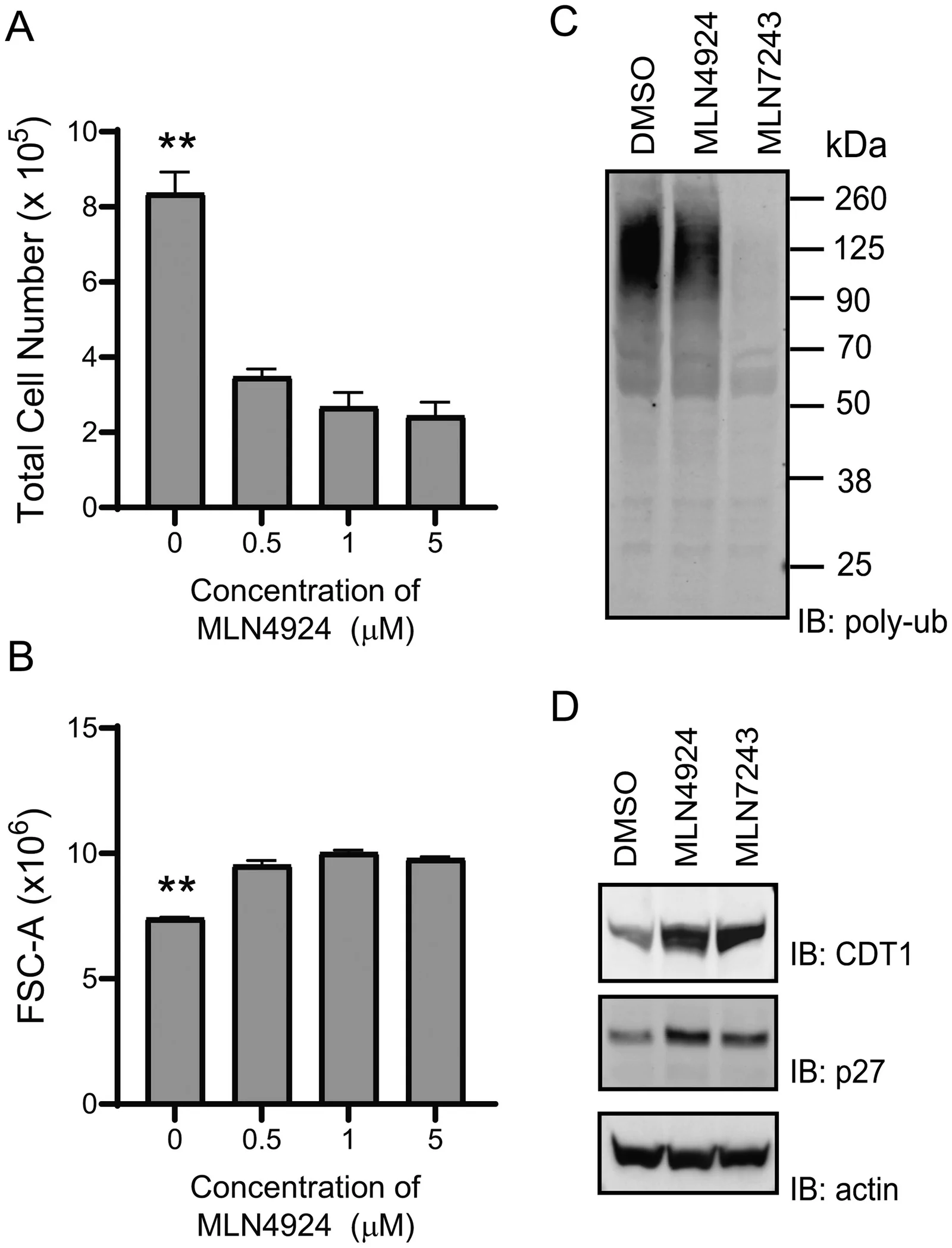
MLN4924 treatment of HeLa cells inhibits CRL function and limits cell division. HeLa cells were treated with indicated concentrations of MLN4924 for 48 h and cells were harvested and counted (A) and analyzed by flow cytometry (B) to measure overall size by forward scatter area (FSC-A). Error bars indicate the SE for 3 technical replicates. The data are representative of 3 independent experiments. **P < 0.01. HeLa cells were treated with either 5 µM MLN4924 or 10 µM MLN7243 for 5 h, then were harvested, lysed, and analyzed by Western blot for either polyubiquitin (poly-ub) levels (C) or the CRL substrates CDT1 or p21 (D). Staining for β-actin was used as a loading control. IB, immunoblot.

MLN492 inhibition of CRL activity should lead to an increase in the levels of cellular proteins which are substrates of CRL E3 ligases, because the substrates will no longer be ubiquitinated and targeted to the proteasome. Cells were treated with 1.0 µM MLN4924 for 5 h and lysed for immunoblot analysis. As a control, cells were also treated with 5 µM MLN7243, a global inhibitor of ubiquitination.^11^ Levels of polyubiquitinated proteins were partially decreased following MLN4924 treatment but were almost completely absent following MLN7243 treatment (Fig. 1C), demonstrating that MLN4924 is not a global inhibitor of protein ubiquitination but can reduce levels of protein polyubiquitination. We also analyzed cell lysates for substrates of CRLs. Levels of p21 and CDT1 increased after MLN4924 treatment (Fig. 1D). The levels of these CRL substrates were similar to those of cells treated with MLN7243, demonstrating that MLN4924 treatment successfully inhibited CRL activity to prevent the ubiquitination and degradation of substrates of CRLs.

### MLN4924 alters the transcriptome to favor autophagy and downregulation of cell cycle transcripts

We next sought to determine how MLN4924 treatment would alter the transcriptome of HeLa cells, after a 48-h treatment with 1.0 µM MLN4924. Differential gene expression analysis indicated that 1,326 genes were significantly upregulated due to MLN4924 treatment compared with DMSO, whereas 1,419 genes were significantly downregulated. Genes with a Benjamini-Hochberg adjusted *P* value of less than 0.05 and a log_2_ fold change of less than −1 or greater than 1 were considered significantly down- or upregulated, respectively (Table S1). Using KEGG pathways annotated for these genes, we conducted a functional enrichment analysis to identify pathways significantly upregulated or downregulated as a result of MLN4924 treatment. As shown in Fig. 2, pathways involved in DNA replication and cell-cycle progression were downregulated following MLN4924 treatment. Conversely, transcripts encoding proteins important for autophagy were upregulated following MLN4924 treatment, consistent with other reports showing induction of autophagy following MLN4924 treatment.^37^^,^^38^

**Figure 2:**
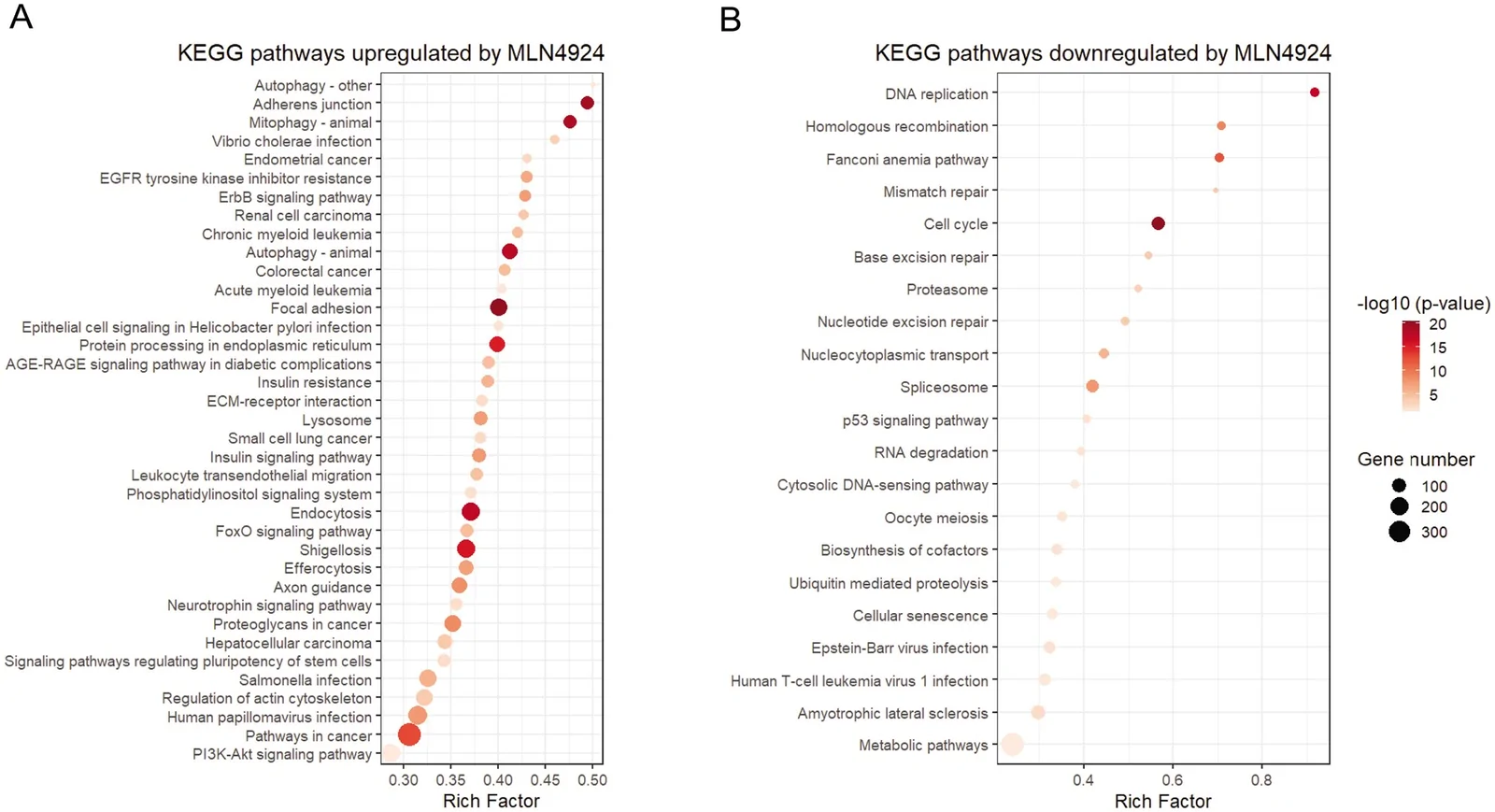
Transcriptome changes to HeLa cells after MLN4924 treatment. HeLa cells were treated with 1 µM MLN4924 (or equivalent volume of DMSO as a control) for 48 h, followed by RNA extraction, RNA-Seq library preparation, and single-end Illumina sequencing. KEGG-pathway enrichment analysis was conducted and separated by pathways whose transcripts increased upon MLN4924 treatment (A) and those that decreased (B). Rich factor was determined and is reported on the *x*-axis. The individual *P* values for each pathway are shown as a color gradient, and the total number of genes is depicted as the size of the circle. ECM, extracellular matrix.

### MLN4924 treatment does not decrease MHC class I expression levels

It is well established that inhibiting either proteasome function or cellular ubiquitination can impair direct MHC class I Ag presentation, leading to a decrease in MHC class I levels at the cell surface. To determine if inhibiting NEDDylation, and thus inactivating CRL ubiquitin ligases, had a similar effect, we cultured HeLa cells in the presence of MLN4924 for 48 h and measured total HLA class I levels using the monoclonal Ab W6/32. As shown in Fig. 3A, total HLA class I levels remained unchanged during the treatment period. As a control, we also included brefeldin A treatment, which globally inhibits trafficking of secretory proteins through the Golgi apparatus. MLN4924 treatment also did not inhibit the secretion of a soluble form of HLA-A*11:01, expressed by HeLa cells. After an initial 5-h treatment with 1 µM MLN4924, the medium was replaced and cells were recultured in medium containing the same concentration of MLN4924 for 4 d. Medium was sampled each day and total secreted HLA was measured via ELISA. As shown in Fig. 3B, soluble HLA-A*11:01 remained relatively consistent, with only a slight decrease by day 4. Taken together, these data indicate that despite inhibiting the action of a large family of ubiquitin ligases, direct Ag presentation was not disrupted.

**Figure 3:**
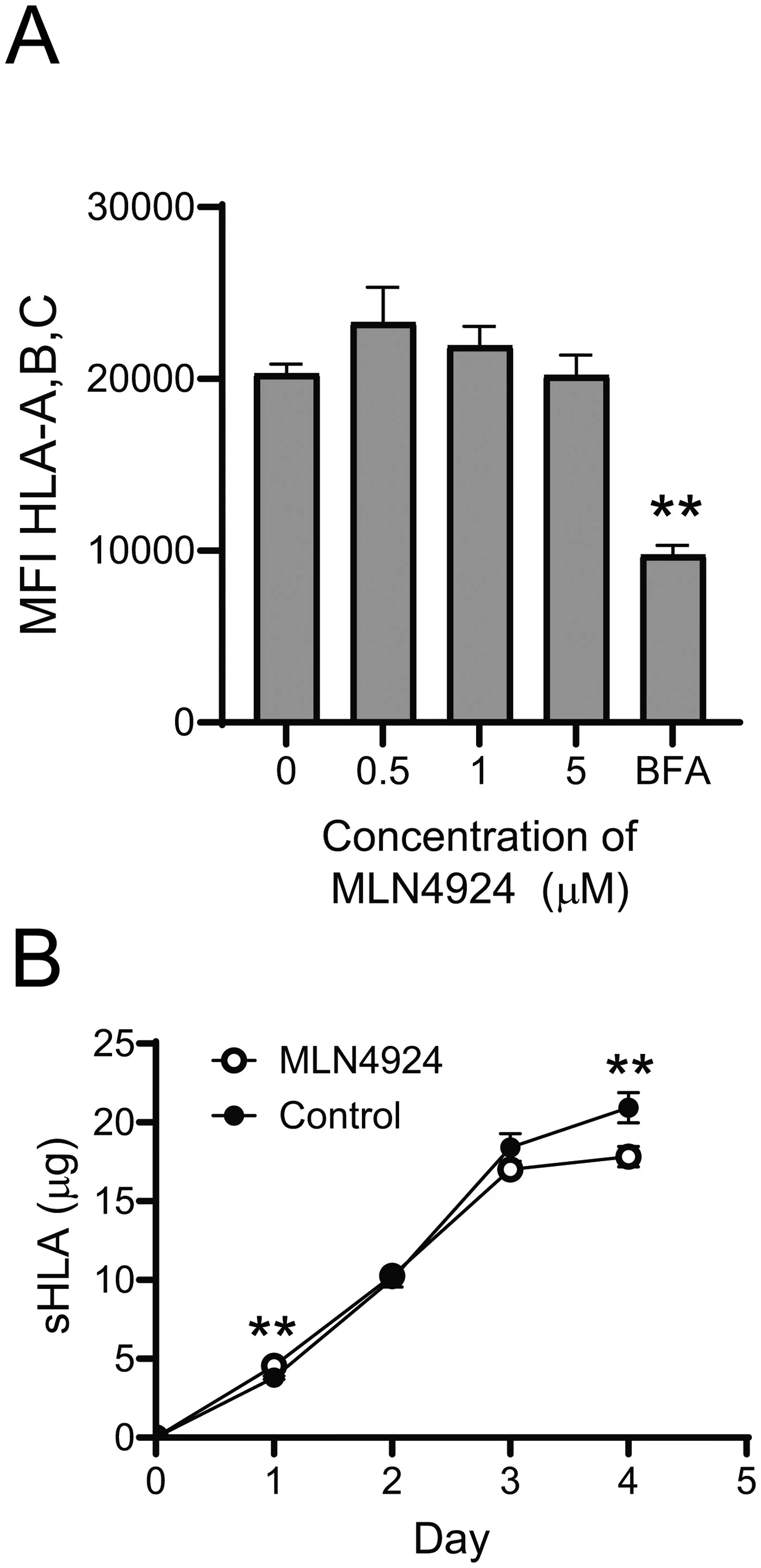
MLN4924 treatment does not globally inhibit direct MHC class I Ag presentation. (A) HeLa cells were treated with the indicated doses of MLN4924 for 2 d and analyzed for total HLA-A, -B, and -C levels using the mAb W6/32. The mean fluorescent intensity of staining, determined using flow cytometry, is reported on the *y*-axis. (B) Secreted HLA-A*11:01 molecules were collected from HeLa/HLA-A*11:01–expressing cells on indicated days after treatment with 1.0 µM MLN4924 and quantified by ELISA. Data are the average of 3 to 4 technical replicates. The data shown is representative of 3 individual experiments. **P < 0.01. BFA, Brefeldin A.

### MLN4924 treatment partially alters the HLA-A*11:01 immunopeptidome

Having established that MLN4924 does not inhibit overall Ag presentation, we sought to determine if inhibiting cellular NEDDylation would alter the identity of peptides presented by MHC class I molecules. Using the secreted HLA system, we treated HeLa/sHLA-A*11:01 cells for an initial 24 h period to allow time for inhibition of cellular NEDDylation. The medium was then discarded to not confound results with sHLA proteins containing peptides derived prior to MLN4924 treatment. Fresh medium containing MLN4924 was added to the cells, and the medium was collected every 3 d over the course of 2 wk. Peptides were isolated from purified HLA molecules and identified using an MS-based immunopeptidomics approach. After removing PTMs and redundant sequences from 3 separate MS runs from treated and untreated cells, we were able to identify a total of 4,482 peptide sequences in untreated cells and 3,223 peptide sequences in MLN4924-treated cells, resulting in the total number of 5,026 stripped, nonredundant peptide sequences (Fig. 4A, Table S2).

**Figure 4:**
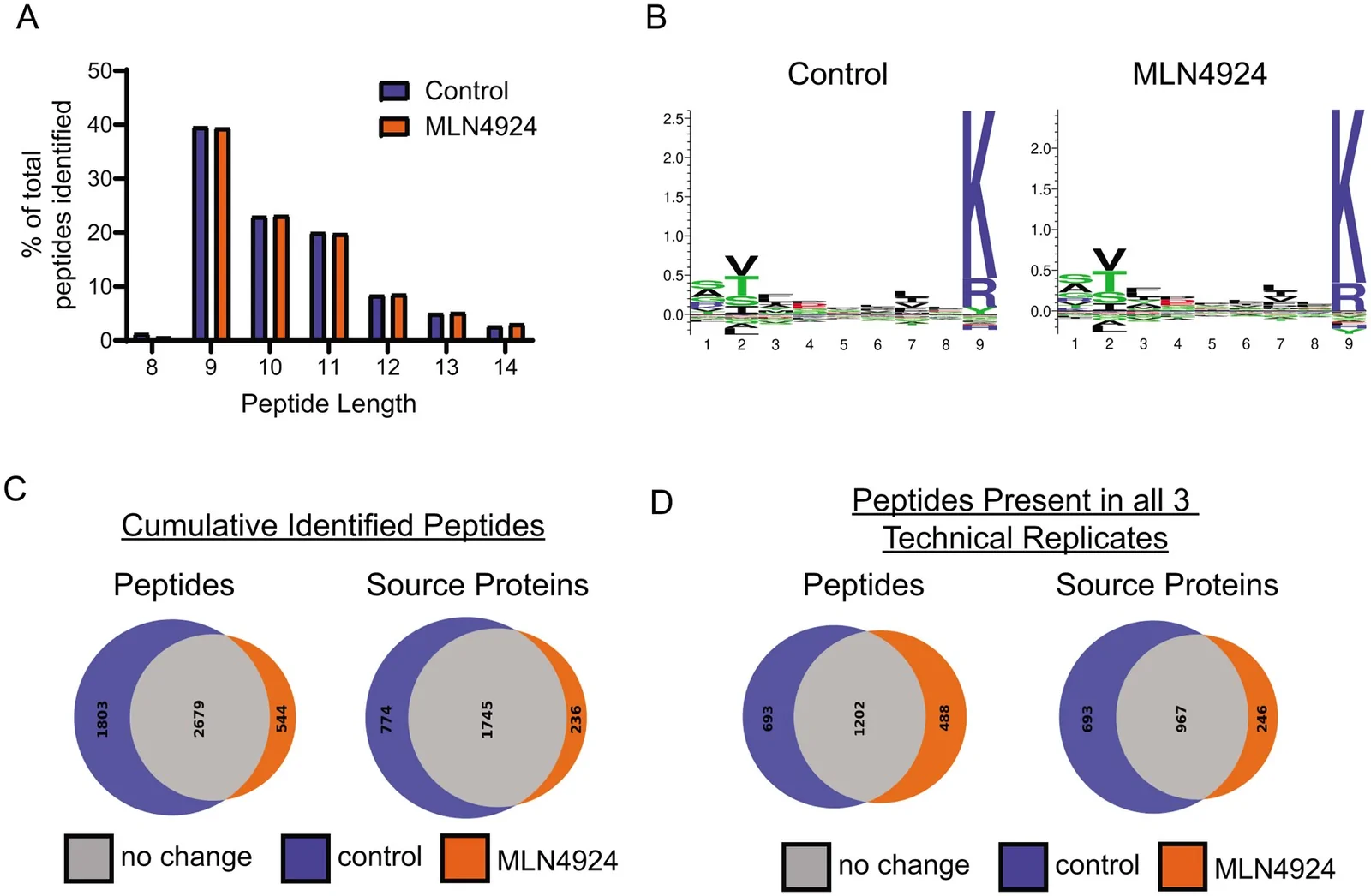
MLN4924 partially alters the immunopeptidome and the identity of source proteins presented by HLA-A*11:01. HeLa/sHLA-A*11:01 cells were cultured with or without 1 µM MLN4924, and secreted HLA-A*11:01 molecules were collected from the cell supernatant by immunoprecipitation. Peptide ligands were eluted, purified by LC, and analyzed by MS/MS. (A) The proportion of peptides with a given amino acid length were determined for untreated and MLN4924-treated cells. (B) We used 9-mer peptide sequences from both untreated and treated cells to determine the peptide binding motif. (C) Venn diagrams show the total number of unique peptides and the source proteins from which they were derived, identified by MS of 3 technical replicates, were segregated based upon appearance after MLN4924 treatment, in control-treated cells, or both. (D) Similar to (C) except the analysis was restricted to only peptides and their source proteins detected in all technical replicates.

Length distribution analysis of HLA-A*11:01-assigned peptides (Fig. 4A) showed an identical pattern in both untreated and MLN4924-treated cells with no significant difference in geometric means (*P* = 0.9814). Supervised clustering of peptide sequences in untreated and MLN4924-treated cells (Fig. 4B) also showed a similar motif consistent with a previously published motif of HLA-A*11:01 peptides (http://mhcmotifatlas.org/home; Gfeller Lab, Ludwig Institute for Cancer Research, University of Lausanne, and the Swiss Institute of Bioinformatics, Lausanne). Among these peptides, 2,679 (53.3% of total peptides) were presented in both untreated and MLN4924-treated samples (Fig. 4C). Certain peptides were uniquely presented either in the presence (*n* = 544 [10.8%]) or absence (*n* = 1,803 [35.9%]) of MLN4924.

Peptides were identified from 2,755 source proteins (Fig. 4C) and, among these proteins, 1,745 (63.3% of the total) were present in both MLN4924-treated and untreated samples. Of source proteins, 744 (28.1%) were presented solely in untreated samples, whereas peptides from 236 proteins (8.6%) were only detected in MLN4924-treated cells. This extensive list of peptides and source proteins was derived from 3 independent MS runs. We therefore used a more conservative approach to analyze the data and analyzed peptides that appeared in all 3 injections for control and MLN4924-treated cells. While this reduced the total number of peptides and source proteins from which they were derived (Fig. 4D), the pattern remained virtually identical. In the restricted dataset, a total of 2,383 peptides were detected in each MS analysis, of which 1,202 (50.5%) were present in both treated and untreated samples. We found 693 peptides that were present only in untreated samples (29.1%), and 488 (20.5%) peptides appeared only after MLN4924 treatment. These data indicate that MLN4924 treatment can partially modulate the identity of peptides presented from different source proteins.

The alterations to the immunopeptidome after MLN4924 treatment could occur due to numerous mechanisms. One simple explanation could be that peptide presentation is directly related to alterations in mRNA transcripts encoding for particular source proteins. Therefore, we compared transcript abundance of source proteins whose presentation was altered after drug treatment. We hypothesized that proteins whose presentation was lost would be reflected in a loss of transcript levels, whereas peptides from source proteins that appeared after MLN4924 treatment would have increased transcript levels. Therefore, we restricted transcriptome analysis to mRNA encoding differentially presented source proteins and visualized the results as a volcano plot (Fig. 5A).

**Figure 5:**
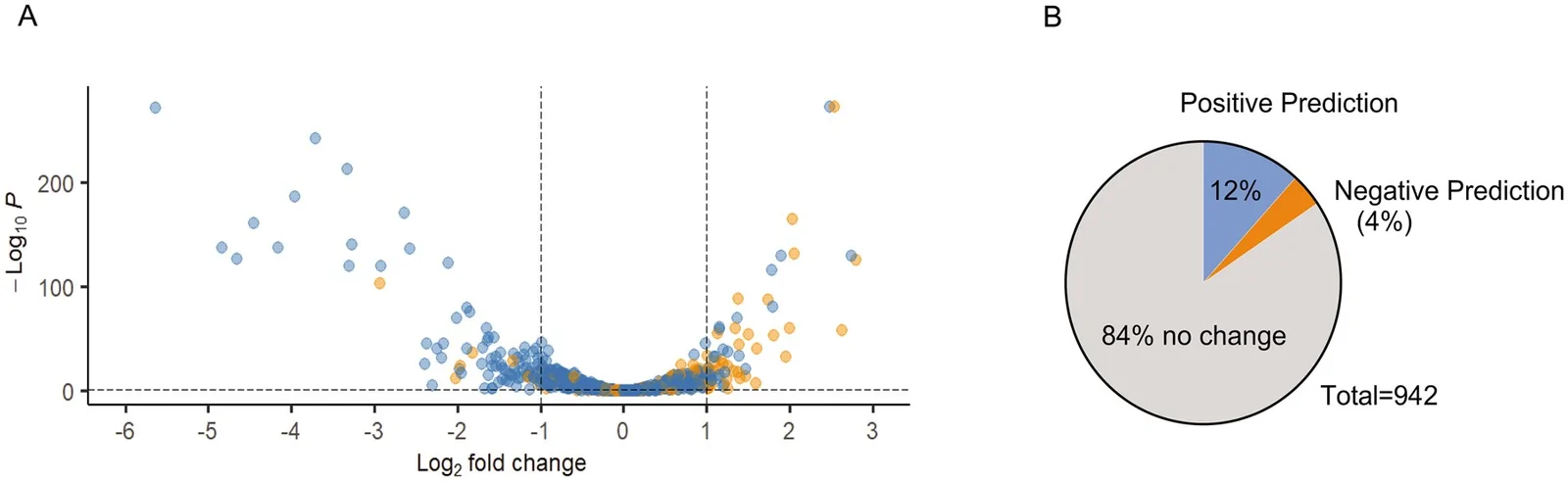
Differentially presented source proteins do not vary in relationship to mRNA abundance. Transcriptome data from Fig. 2 were analyzed to determine changes in mRNA levels for source proteins whose peptide presentation changed after MLN4924 treatment. (A) Blue dots represent transcripts encoding source proteins that were no longer presented after MLN4924 treatment; orange dots represent transcripts encoding source proteins that appeared after treatment. (B) The fraction of transcripts predicted to change (ie either decrease if peptides from the source protein are no longer presented or increase if the peptides from a source protein appear) and the fraction of transcripts with the opposite prediction are shown along with transcripts whose levels did not appreciably change.

Of the 942 transcripts detected that corresponded to differentially presented source proteins, 796 (85%) did not appreciably change (<1 log_2_-fold change), despite the gain or loss of peptide presentation (Fig. 5B). Of the remaining ∼15% of transcripts that did change, ∼12% followed a predicted pattern—that is peptides appeared when transcripts increased and vanished when transcripts decreased. However, there were peptides presented from proteins whose transcript levels moved in opposing directions (negative prediction). Some peptides appeared despite lowered transcript levels and others disappeared when transcript levels increased. These data indicate that changes to the peptidome after MLN4924 treatment could not be predicted by measuring changes to the cellular transcriptome.

### Peptides from CRL substrates are presented after MLN4924 treatment

MLN4924 treatment is known to inhibit CRL function and thus prevent the ubiquitination of particular substrates; therefore, we searched our list of HLA-A*11 peptides derived from source proteins (Table S3) that were either known substrates of CRL ligases or had been reported to increase after MLN4924 treatment.^39–49^ We suspected that MLN4924 treatment would prevent the presentation of peptides from these substrates because they would no longer be ubiquitinated and degraded by the proteasome. We found peptides from 108 source proteins that are known or suspected substrates of CRLs (Fig. 6A). Of these, peptides from 68 source proteins were presented independently of MLN4924 treatment. Peptides from only 33 CRL substrates were lost after MLN4924 treatment, and peptides from 7 source proteins appeared. Contrary to our hypothesis, peptides from the majority of CRL substrates were still presented after inhibiting CRL activity.

**Figure 6:**
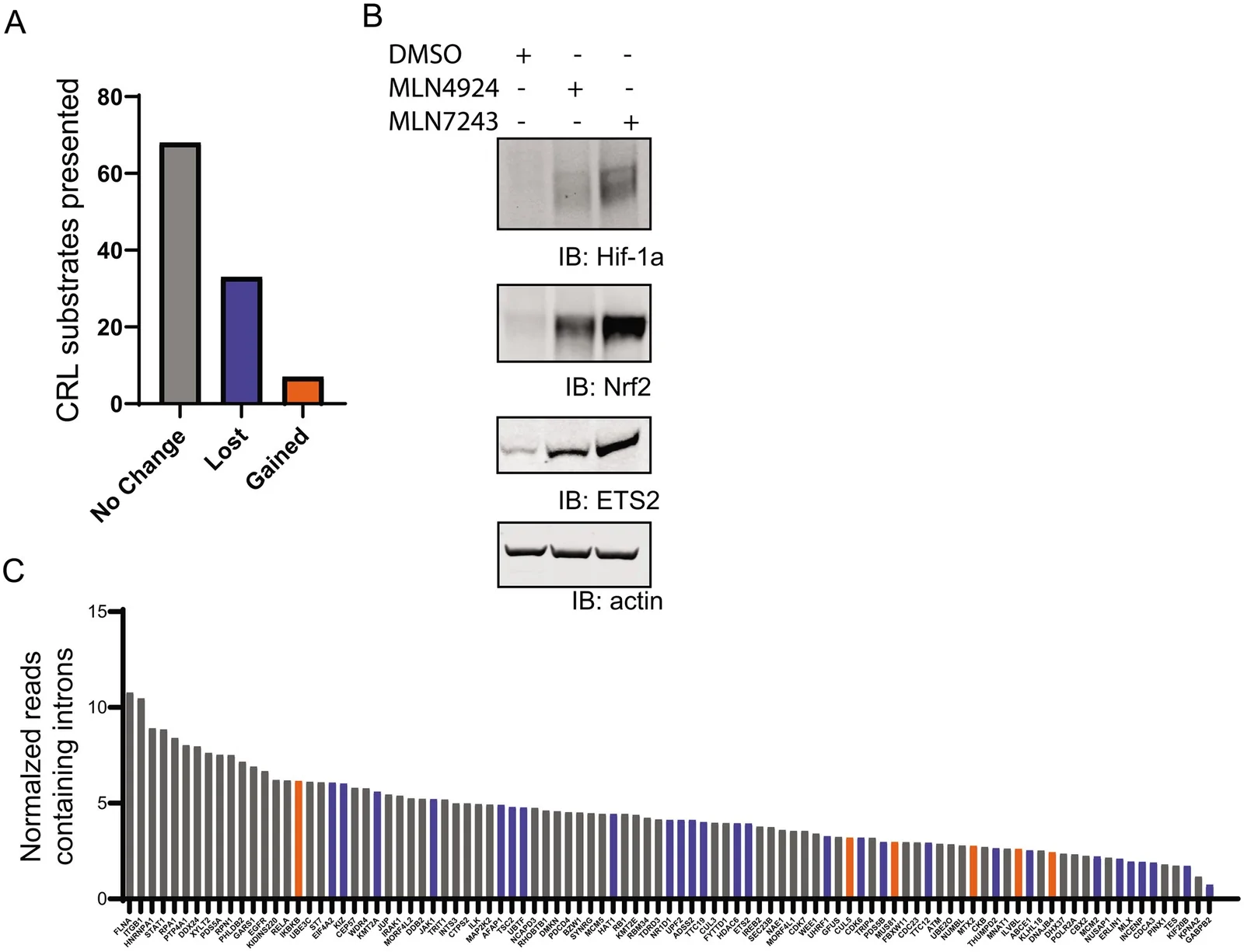
Peptides from CRL substrates after MLN4924 treatment. Approximately 100 proteins that yielded peptides for presentation and are known substrates of CRLs were examined to determine if MLN4924 treatment prevented peptide presentation. Results are tabulated in (A), with gray bars denoting substrates presented irrespective of MLN4924 treatment, blue bars indicating those proteins no longer presented after MLN4924 treatment, and orange bars denoting proteins presented only in the presence of MLN4924. (B) Western blot analysis of the indicated CRL substrates after 5 h of treatment with MLN4924 or MLN7243. Actin was used as a loading control. (C) Transcripts encoding CRL substrates were analyzed for the presence of intron sequences, and the relative number of sequences is plotted for each protein. The *y*-axis value is log2 counts per million reads. The color of bars are the same as in (A). IB, immunoblot.

There could be numerous reasons for the ongoing presentation of peptides from source proteins that would no longer be degraded after CRL inactivation by MLN4924 treatment. One possibility is CRL-independent ubiquitination. To test this, we compared total cell lysates from MLN4924- and MLN7243-treated cells to compare the abundance of proteins after either CRL inactivation or global inactivation of ubiquitination, similar to data shown in Fig. 1. Unlike p21 and CDT1, which accumulated to similar amounts, levels of other proteins, such as HIF1α, ETS-2, and Nrf2, were much higher when ubiquitination was inhibited (Fig. 6B), compared with inhibition of CRLs, indicating that certain CRL substrates can be ubiquitinated by other means. In the case of HIF1α, the HLA-A*11:01 peptide SVMRLTISY was presented in both untreated and MLN4924-treated cells. These data suggest that alternative ubiquitination pathways exist that can target known or suspected CRL substrates for ubiquitination and proteasomal degradation, resulting in ongoing peptide presentation when CRLs are no longer active.

Another possible explanation for the presentation of peptides from CRL-targeted proteins during MLN4924 treatment could arise from mRNA transcripts bearing intronic sequences. That MHC-bound peptides can be generated from introns^50^^,^^51^ has been confirmed using model peptides both in vitro and in vivo^52^^,^^53^ and analysis of large peptide databases.^54^ The translated protein of a mis-spliced mRNA will likely result in a protein that folds into a different conformation than that of a properly translated protein. The alteration of protein structure could alter its recognition by CRL E3 ligases and lead to degradation by a different pathway. Therefore, we queried our transcriptome data for the presence of intronic sequences using STAR and Salmon and normalized expression of intron-containing sequences to the total library size. We restricted our analysis to the set of transcripts encoding suspected CRL substrates, which yielded detectable peptides bound to HLA-A*11:01. As shown in Fig. 6C, peptides that were lost after MLN4924 treatment were more likely to occur from transcripts with lower intron numbers, compared with peptides presented in both treated and MLN4924 cells. A ranking of the transcripts by intronic abundance demonstrated that transcripts encoding proteins dependent on CRL function for peptide presentation were skewed lower than transcripts that encoded peptides presented independently of MLN4924 treatment. Of the top 20% transcripts most likely to contain introns, only 1 encoded a source protein exclusively presented in the absence of MLN4924. At the other end of the spectrum, 8 source proteins were no longer presented during MLN4924 treatment, representing nearly 40% of the source proteins in this region. These data indicate that intron-containing mRNA sequences could generate translation products, which can be degraded and presented in the absence of functional CRLs brought about by MLN4924 treatment.

The immunopeptidomic data presented thus far were derived from secreted HLA-A*11:01 molecules produced in HeLa cells. To determine if alterations to the immunopeptidome extended to other HLA allotypes and in different cells, we isolated total HLA class I proteins from the human glial cell line U87-MG after MLN4924 treatment. Similar to HeLa cells, MLN4924 prevented cell division and did not alter overall MHC class I levels at the cell surface after a 2-d MLN4924 treatment (Fig. 7A, B). After cell lysis, HLA proteins were isolated by immunoprecipitation with the mAb W6/32 and peptide ligands identified by MS. As shown in Fig. 7C, MLN4924 treatment again only partially altered the immunopeptidome. A total of 8,210 nonredundant peptide sequences were identified from 3 separate MS runs for both MLN4924-treated and untreated samples. Similar to the results from secreted HLA-A*11:01 in HeLa cells, approximately 53.8% of the peptides were present with or without exposure to the drug. After treatment, 2,013 of the peptides (24.5%) were no longer detected, whereas 1,779 (21.7%) appeared. Based on peptide sequence, we classified peptides as being presented by HLA-A*02:01, HLA-B*44:02, or HLA-C*05:01 proteins, which represent the reported haplotype of U87-MG cells. Though there are some slight variations in absolute numbers, the general pattern of a minor alteration to the immunopeptidome upon drug treatment remained consistent (Fig. 7C).

**Figure 7:**
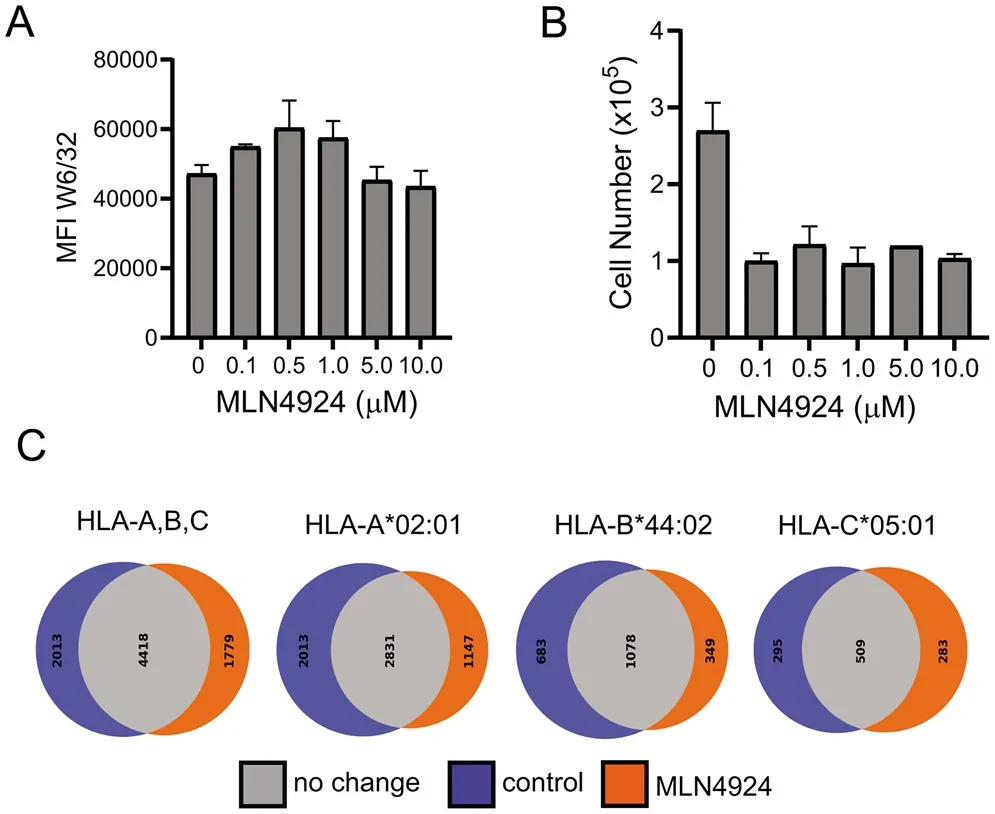
MLN4924 partially alters the immunopeptidome and the identity of source proteins presented by HLA proteins in U87-MG cells. Approximately 1 × 10^5^ U87-MG cells were treated with indicated concentrations of MLN4924 for 48 h and analyzed for total cell-surface HLA–MHC class I (A) and total cell number (B). (C) U87-MG cells were cultured for 48 h in the presence or absence of 1 µM MLN4924. Cells were then lysed and total HLA–MHC class I proteins were isolated by immunoaffinity chromatography. Peptide ligands were eluted and analyzed by MS. Peptides were classified as appearing after MLN4924 treatment (orange) or present in control-treated cells only (blue). Peptides or proteins present in both treated and untreated samples are gray. Peptides were further classified by their binding motif to the HLA allotypes of U87-MG cells.

## Discussion

Future treatments in oncology will no doubt seek to combine T cell–based immunotherapies with powerful chemotherapies to combat cancer. However, care should be taken when combining medications, because the additive effects may not materialize if chemotherapies interfere with direct MHC class I Ag presentation. For instance, the addition of an ICI to enhance T-cell activation may be negated if the chemotherapeutic agent in question inhibits Ag presentation, making the tumor invisible to T cells. In addition to considering global MHC class I Ag presentation, changes to the immunopeptidome of the tumor cells after chemotherapeutic treatment also should be considered, especially if tumor vaccination strategies are in place. Here, we report on the impact of MLN4924, a potentially valuable anticancer compound, and its effect on direct MHC class I Ag presentation. It is especially important to consider the influence on the immunopeptidome, because MLN4924 treatment inhibits cellular NEDDylation, which, in turn, inhibits the action of several hundred E3 ubiquitin-ligating enzymes, which target numerous proteins for degradation and, by extension, antigenic peptide generation. If NEDDylation inhibitors are to be used as chemotherapeutic agents, it will be important to determine their utility when combined with immunotherapy.

Our results indicate that MLN4924 treatment causes a partial alteration to the immunopeptidome of tumor-cell lines. Although changes were detected, it is worth noting that more than 50% of the peptides detected were still present after MLN4924 treatment. This pattern was consistent when examining either total cellular HLA proteins or secreted HLA proteins. Analyzing peptide ligands from both types of HLA molecules is valuable. Secreted HLA molecules allow for precise kinetic control over peptide production, which is essential when considering how drugs and small molecules affect Ag presentation. After treating cells for just a few hours, tissue culture media can be changed and all peptide-MHC complexes generated prior to drug treatment will be removed from downstream analysis. Thus, only peptides arising after drug treatment are analyzed. In contrast, cell-associated MHC class I molecules will retain peptides created prior to drug treatment until peptide-MHC complexes completely turnover, which may take hours to days; therefore, peptides identified may not reflect the impact of the drug on Ag presentation. However, secreted HLA molecules can only acquire peptides in the ER and not through alternative pathways, such as recycling,^55^ TAP-independent pathways,^56^ or in endosomal compartments on which certain HLA allotypes rely.^57^^,^^58^ The similar pattern we observed in 2 different cell types, different HLA allotypes, and with both secreted and cell-associated HLA suggest that MLN4924 treatment will consistently have a partial effect on the identity of the immunopeptidome, though work will need to validate this claim.

Changes in the immunopeptidome mirrored changes to the identity of source proteins from which peptides were derived. Presentation of peptides from approximately two-thirds of the proteins that provided peptides to the immunopeptidome were unchanged after MLN4924 treatment. Although changes were detected, the majority of both individual peptides and the proteins they were derived from could still potentially be identified by cytotoxic T cells. Though pevonedistat (MLN4924) is designated as a Breakthrough Therapy by the US Food and Drug Administration for the treatment of a variety of blood cancers, phase 3 trial results were not encouraging.^59^ Because our data suggest that MLN4924 has a minimal impact on direct Ag presentation, clinicians may want to consider combining ICI therapy with pevonedistat for cancer treatment.

How might we explain the changes to the immunopeptidome we observed? The simplest explanation could be that the cellular response to MLN4924 treatment alters the mRNA transcriptome and, by extension, the peptides presented. A relationship between the immunopeptidome and transcriptome of cells has been tested in several studies but often has yielded conflicting results.^60–68^ Our study offer a relatively simple approach to this question by simply comparing the immunopeptidome and transcriptome after drug treatment, whereby direct changes can be measured without having to rely on other comparisons, such as healthy tissues to cancerous tissues. We were only able to “predict” approximately 11% of the observed alterations to the immunopeptidome; that is, either a peptide appearing as its source-protein transcript increased or a peptide was lost as the source-protein transcript decreased. Most of the observed changes to the immunopeptidome occurred without a change to the source-protein transcript (∼85%), and some alterations to peptide ligands contradicted our hypothesis completely. Although transcriptome analysis may be a simple and cost-effective way to predict changes to the immunopeptidome after drug treatment, our data suggest caution against using changes to the transcriptome to predict changes to the immunopeptidome.

Another likely explanation for changes to the immunopeptidome relates to the mechanism of action of MLN4924, which inhibits NEDDylation of CRLs, thus preventing ubiquitination and degradation of CRL substrates. If the substrates of CRLs are no longer degraded via the proteasome, then peptides liberated by degradation would be unavailable for presentation. Therefore, we compiled a list of known or potential CRL substrates whose peptides were detected bound to HLA-A*11:01 proteins. By combining articles reporting on examination of protein accumulation after MLN4924 treatment with numerous reports identifying CRL substrates by traditional biochemical methods and then cross-referencing the list with the source proteins described in this report, we were able to generate a list of approximately 100 source proteins that are CRL substrates and from which we detected HLA-A*11:01–bound peptides. Contrary to our supposition, peptides from only ∼25% of these substrates were lost after MLN4924 treatment. There are several possible reasons for this surprising finding that point to some interesting aspects of cell biology.

One explanation could be that these substrates are degraded in a CRL-independent manner. Although we found that some proteins accumulate to a similar extent when either CRL activity or global ubiquitination was inhibited, this did not hold true for all proteins. For instance, HIF1-α was much more abundant in cell lysates after ubiquitination inhibition, suggesting that peptides derived from this substrate can be degraded after ubiquitination by other E3 ligases. One could also imagine other pathways of destruction, such as ubiquitin-independent proteasome degradation or even autophagy as routes for a protein’s destruction and antigenic peptide generation.

The source of peptides may also be defective ribosomal products, which may be misfolded forms of the source protein or be “defective” in some other way and, therefore, may not resemble the protein substrate as would be seen by the CRL. It may be possible for an unfolded form of a protein, which contains the MHC-associated peptide amino acid sequence, to be degraded by different proteases or ubiquitinated by different E3 ligases. We examined this possibility by looking for intronic sequences in the transcripts encoding peptide-producing source proteins normally subject to CRL ubiquitination. A clear trend emerged that suggested peptides from CRL substrates presented independently of MLN4924 treatment were more likely to contain intronic sequences of RNA compared with those substrates that did not yield peptides upon MLN4924 treatment (Fig. 6). One could envision how the inclusion of extra nucleotides in the mRNA could generate mutated forms of the protein, which may be rapidly degraded following their generation. If the translated intron generated a premature stop codon, then it is entirely possible that the resulting polypeptide would not be recognized by its canonical CRL, and thus peptide generation would not require CRL activity. Many cryptic peptides and neoantigens presented by MHC class I molecules are derived from intron-containing transcripts, lending credence to this hypothesis.^54^^,^^69^

In summary, the first-in-class NEDDylation inhibitor MLN4924 appears suitable for combinatorial cancer treatments using immunotherapies such as ICIs, because MLN4924 treatment does not appear to alter direct MHC class I Ag presentation and only has a minor effect on the composition of the immunopeptidome. Furthermore, MLN4924 treatment reveals that there are multiple pathways for a given protein to be degraded into antigenic peptides, perhaps due to either the nature of the precursor protein or to different E3 ubiquitin ligases, which can target the same protein for degradation. Further exploration of this cellular biology is warranted both for clinical use and for understanding the nature of MHC class I Ag presentation.

## Supplementary Material

vkag258_Supplementary_Data

## Data Availability

The raw mass spectrometry data from soluble A*11:01–transfected HeLa cells and U87MG cells used in this study have been deposited in the Center for Computational Mass Spectrometry (MassIVE) under accession number MSV000102347.
